# Bibliometric Analysis of Global Scientific Literature on Mumps Vaccines

**DOI:** 10.7759/cureus.88976

**Published:** 2025-07-29

**Authors:** Debabrata Tripathy, Debkumar Pal, Pravin M Pisudde, Manish Taywade, Asmita Patnaik

**Affiliations:** 1 Community Medicine and Family Medicine, All India Institute of Medical Sciences, Bhubaneswar, IND; 2 Community Medicine and Family Medicine, All India Institute of Medical Sciences, Kalyani, IND; 3 Community Medicine, All India Institute of Medical Sciences, Hyderabad, IND; 4 Community and Family Medicine, All India Institute of Medical Sciences, Bhubaneswar, IND

**Keywords:** bibliometrics, mumps vaccine, primary prevention, systematic literature review, vosviewer

## Abstract

Mumps is a vaccine-preventable disease commonly affecting children. Mumps vaccines (MuVac) are currently marketed in polyvalent forms. This study used bibliometric analysis to study the pattern of performance of articles, authors, and journals, and to disclose gaps and constituents of research on MuVac. The study was conducted using the Scopus database. Different keywords related to MuVac were identified, and a search was conducted. All studies published in any language between 1972 and 2021 were included in the analysis. The data was exported, a basic descriptive analysis was done, and an analysis was done using the Biblioshiny R software package. VOSviewer software was used to construct and visualize the bibliometric network. A total of 699 publications on MuVac were gathered, with an average citation of 33.87. The maximum number of 66 articles was published in 2015. Using Bradford's law, the core zone contained 14 journals with 232 articles. Top documents were analyzed based on top local citations, global citations, and the highest locally cited references. Best authors based on the highest number of studies published, citations, and author impact were analyzed using h-, g-, and m-induces. Journal-level performances were analyzed in the headings of top sources, the journal with the highest total number of citations. The study using bibliometric analysis has helped to identify top authors, journals, institutions, and countries in the field of research on MuVac. This study has summarised the pattern and trends of research activity on MuVac and will help provide valuable perspectives and direction for future studies. Newer software like R and VOSviewer helps conduct bibliometric analysis and data visualization.

## Introduction and background

The mumps virus is an enveloped, single-stranded RNA virus classified within the paramyxoviridae family [1]. This group also includes the parainfluenza virus [2]. The mumps virus is antigenically stable and does not undergo genetic shifts like the influenza virus [3]. At least 12 genotypes are defined based on the small hydrophobic (SH) gene sequence [4]. Mumps is a vaccine-preventable viral disease usually affecting children, and natural mumps infection has been considered to give lifelong immunity [5,6]. The primary mode of transmission of the mumps virus is through the respiratory or oral route via infected respiratory droplets or secretions [7]. There are several reported complications of mumps, among which aseptic meningitis and orchitis are the most common [3]. Mumps has been neglected compared to other childhood infections like measles due to the shorter infection duration and fewer reported complications [8]. Globally, the incidence rate of mumps was almost 1,000,000 population, with the occurrence of many unreported cases. The mild cases often remain unnoticed, while severe cases may lead to complications in the form of pneumonia or sensorineural deafness [9].

The mumps vaccine was first developed and used in the United States in 1950 [5]. Live attenuated vaccine for mumps has been developed and used in the United States and the Soviet Union since the 1960s [3]. Different strains of the mumps virus were used to develop vaccines. The United States and the Soviet Union used the Jerry Lynn strain and the Leningrad-3 strain, respectively. The most popularly used strains worldwide are Jerry Lynn and Urabe Am, followed by other strains [10]. Mumps vaccines were initially produced as a monovalent vaccine, which contained only the mumps component, but the currently manufactured mumps vaccines are trivalent vaccines containing measles and rubella components along with the mumps component [11]. The known combinations of measles, mumps, and rubella (MMR) vaccines have been developed worldwide since the mid-1960s, and currently, more than 120 countries are using them [3,12]. The measles-mumps rubella-varicella (MMRV) vaccine has been available in the open market since the mid-2000s and was developed based on the existing MMR and varicella vaccines [13]. A clinical study found that giving a single dose of the Jeryl Lynn strain mumps vaccine causes seroconversion in about 80-100% of cases [14]. Multiple studies conducted globally have found that a two-dose regimen results in a very high seroconversion rate [15]. The duration of vaccine-induced immunity has not been well documented, but detectable specific mumps virus-neutralizing antibodies correlate with resistance to clinical infection [6]. The effectiveness of two doses of MMR immunization is under question, with several studies giving evidence of a waning of immunity with time, as several sporadic mumps outbreaks have been reported even after good two-dose vaccine coverage [16,17]. Currently, almost 100 countries include at least one dose of the mumps vaccine, mainly in the form of MMR, with most of them belonging to high-income countries [18].

Enhancement of knowledge regarding the mumps virus, vaccines, and concerns over vaccines like safety, immunogenicity, adverse events following vaccination safety and efficacy will help intervene in the spread of mumps virus infection [15]. In recent times, the popularity of bibliometric analysis has increased. Researchers and scholars are using bibliometrics as an emerging tool to study the pattern of emerging articles, journal performance, and author collaboration patterns, and disclose gaps [19]. Mathematical, computational, statistical, and other methodologies are used in bibliometric analysis to extract the data needed [20]. A bibliometric analysis was conducted to review the literature on viruses like hepatitis B, rubella, and new-age pathogens like Zika and COVID-19 [18]. However, no comprehensive bibliometric analysis has yet been done on the mumps virus, vaccine, or disease. This study aimed to evaluate the position of all research in the literature on vaccination related to mumps and to describe and depict the characteristics and trends using a bibliometrics approach.

## Review

Materials and methods

We conducted this bibliometric study using the SciVerse® Scopus database. Scopus is the largest database related to medical literature, indexing over 90 million records, including over 49 million patents, 290,000 books, and 27,000 active scholarly titles. It is a paid subscription-based database usually available through institutional access. There is a provision of one month of free, complimentary access for peer reviewers after completing one peer review for any Scopus-indexed journal [20]. It is an approved database for conducting bibliometric analysis as it provides information on citation and author metrics. Different keywords related to mumps immunization or vaccination were recognized, and a detailed search strategy was developed. The search used Boolean operators 'AND' and 'OR' to identify the articles containing selected keywords. All studies published in any language from the 1st January 1972 till the 31st December 2021 were included in the analysis (Table 1). The search was conducted on the 31st March 2024.

**Table 1:** Details of the search strategy for selecting the appropriate article

Heading	Description
Topic	Mumps vaccine
Coverage & scope	Scopus
	Articles, reviews, editorials, research letters, letter, note, review
	January 1972-December 2021
String & keyword search	( TITLE-ABS-KEY ( mumps ) AND TITLE-ABS-KEY ( vaccin* OR immun* OR safety OR aefi OR protection OR prevention OR injection OR cold AND chain OR antibody OR antigen OR efficacy OR effectiveness OR adverse AND event OR adverse AND effect ) )
String & keyword search	N=699
	With no language barrier (non-English=40)

The search results obtained from Scopus were exported in comma-separated values (CSV) format. The exported data sheet contained several data such as author details, citation, author country, title, doi, abstract, etc. A basic descriptive analysis identifying the number of documents, citations received, and year of publication of the exported details was conducted using Scopus. The CSV file was imported into the Biblioshiny package of R Studio to convert it into an R-enabled format. The biblioshiny package of R software was used to analyze the R software-enabled CSV file, and the data file was uploaded to the package. The uploaded data was used to analyze data in further headings, as mentioned in Table 2.

**Table 2:** Data was analysed using Biblioshiny package of R software

Features of mumps vaccine-related study	Main information
Yearly distribution of documents published	Annual scientific production
Distribution of document types	Bradford's Law
Top locally most cited documents	Most cited local document
Top globally most cited documents	Most cited global document
Top locally cited references	Most local cited references
Top authors with highest number of studies	Most relevant author
Top authors with highest number of citations	Authors' local impact
Top authors with highest number of impact factor	Authors' local impact
Countries with highest scientific production	Corresponding authors country
Countries with highest number of citations	Most cited countries
Top sources with documents published	Most relevant sources
Top sources with highest local citations	Sources' local impact
Sources with highest impact factor	Sources' local impact
Top keywords	Most frequent words
Top country collaboration	Countries collaboration world map

The data sheet extracted from Scopus in CSV format was uploaded to VOSviewer (Leiden University, Leiden, The Netherlands) to construct and visualize the bibliometric network. These bibliometric networks included journals, researchers, and the country of publication, and they were established based on citation, co-citation, etc.

Ethical Approval

Ethical approval was not required for the analysis of the previously published research. We have not used any data from any of the human participants, and we followed the International Committee of Medical Journal Editors (ICMJE) guidelines regarding the same.

Results

Features of Mumps Vaccine-Related Studies

The primary information regarding the studies conducted on the mumps vaccine on Scopus from 1972 to 2021 is demonstrated in Table 3.

**Table 3:** Information about studies on the mumps vaccine on Scopus from 1972 to 2021

Description	Results
Time span	1972-2021
Sources (journals, books, etc.)	357
Documents (articles, reviews, editorials, and research letters)	699
Annual growth rate (in %)	6.38
Document average age	14.2
Average citations per document	33.87
References	25,008
Document contents	
Keywords plus (ID)	5876
Authors' keywords (DE)	1342
Author & author collaboration	
Authors	2947
Authors of single-authored documents	88
Authors of multi-authored documents	2859
Single-authored documents	91
Co-authors per document	5.05
Document types	
Articles	471
Book chapter	7
Conference paper	12
Editorial	4
Erratum	1
Letter	7
Note	8
Review	182
Short survey	7

Using Scopus, a total of 699 publications were gathered. The average number of citations per publication for 357 sources (articles, reviews, editorials, and research letters) was 33.87. There was a total of 2947 authors, which included 2859 multi-authored and 88 single-authored documents. Out of 699 documents published during the period on the mumps vaccine, 471 documents were articles, 182 were reviews, seven were book chapters, 12 were conference papers, and seven were short surveys.

Distribution of Year of Publication of Mumps Vaccine-Related Studies

**Figure 1:**
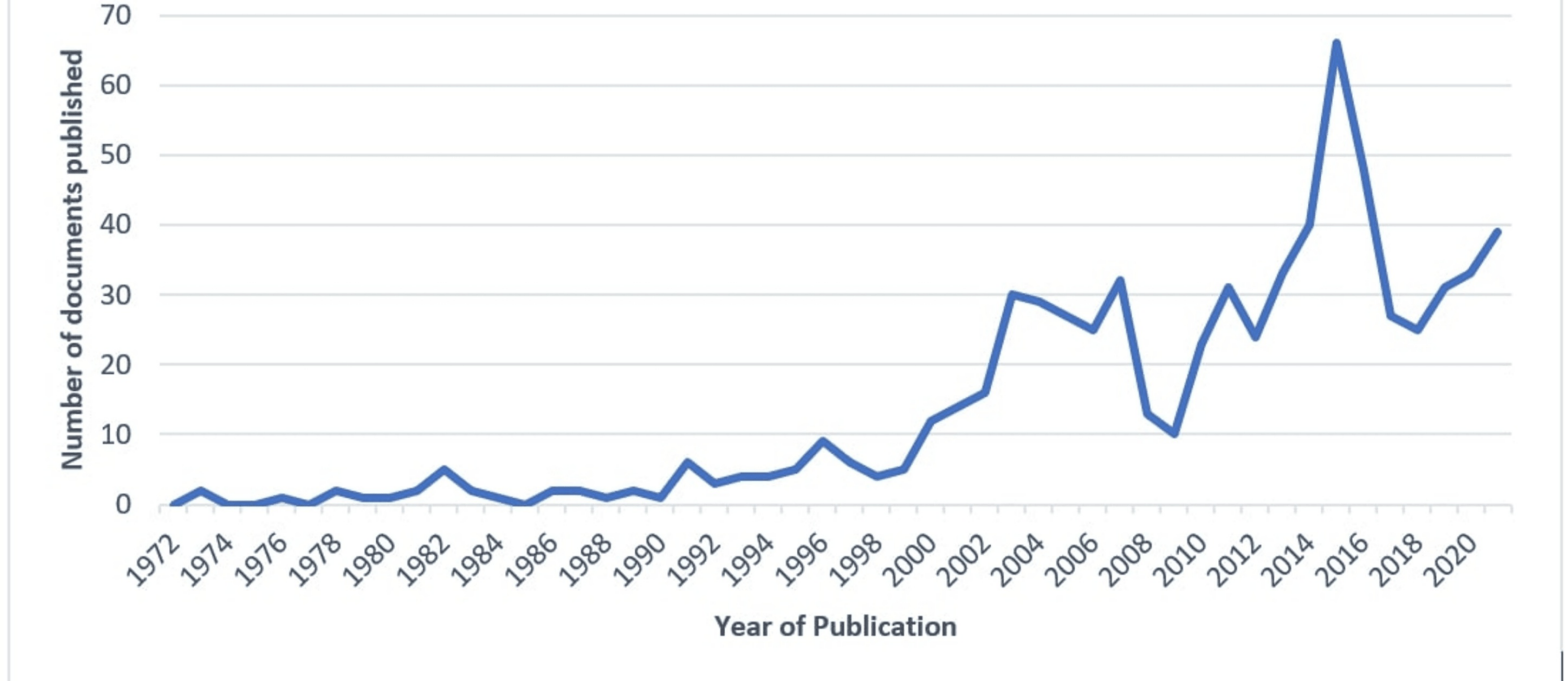
Number of mumps vaccine-related documents published per year

The maximum number of documents published in 2015 was 66 (Figure 1).

No documents were published in 1972, 1974, 1975, 1977, or 1985. There was only one publication in 1976, 1979, 1980, 1984, 1988, and 1990. There is a rise in the number of published documents over time.

Distribution of Document Types of Mumps Vaccine-Related Studies

**Figure 2:**
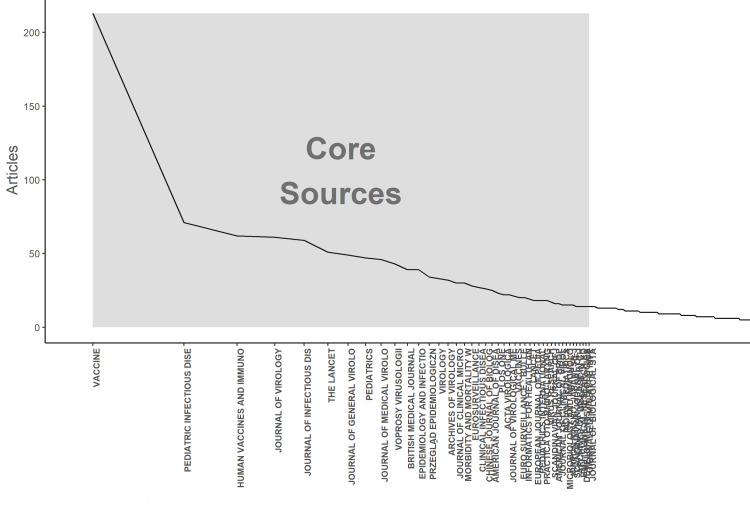
Distribution of source by Bradford's law

Using Bradford's law, 14 journals with 232 citations and 113 journals with 237 citations were present in the core zone and core + zone 2, respectively (Figure 2).

Characteristics of the Most Cited Studies on the Mumps Vaccine

**Figure 3:**
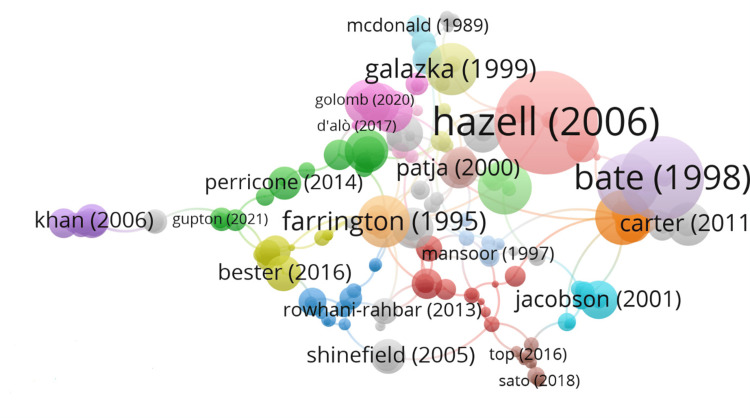
Most cited documents on the mumps vaccine

The top 100 most-cited articles were arranged in descending order by citation frequency. The top three most cited papers were further analyzed for their citations. The top three locally cited documents were by Farrington et al. [21], Varricchio et al. [22], and Peltola et al. [23], with 25, 23, and 23 local citations and 280, 312, and 183 global citations, respectively. The top three globally cited documents were by Hazell et al. [24], Bate et al. [25], and Klein et al. [26] with 1305, 817, and 601 total citations and 68.68, 30.26, and 40.07 total citations per year, respectively (Figure 3).

The top three locally cited references were Chen et al. [27], Farrington et al. [28], and Nakayama et al. [29], with eight citations each.

Distribution of Authors of Mumps Vaccine-Related Publications

**Figure 4:**
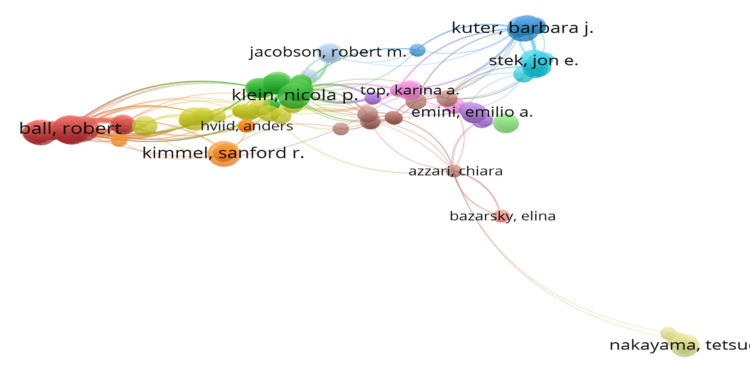
Outstanding authors of publications on mumps vaccine

Robert Chen, Frank DeStefano, and Robert Ball were the top three authors with the highest number of studies, with 10, eight, and seven articles, respectively. The authors with the highest number of global citations were Lorna Hazell, Saad AW Shakir, and Frank DeStefano, with 1305, 1305, and 1211 citations, respectively (Figure 4). 

The top three authors on the basis of impact factor were Robert Chen, Frank DeStefano, and Barbara J Kuter with an h-index of 10, eight, and seven; a g-index of 10, eight, and seven; m-index of 0.294, 0.32, and 0.35; and total number of citations of 1001, 1211, and 226, respectively. William Joseph Bellini, Paul A Rota, and Heikki Peltola started publishing in 1991, 2000, and 1985, respectively.

Impact of the Source of Mumps Vaccine-Related Studies

**Figure 5:**
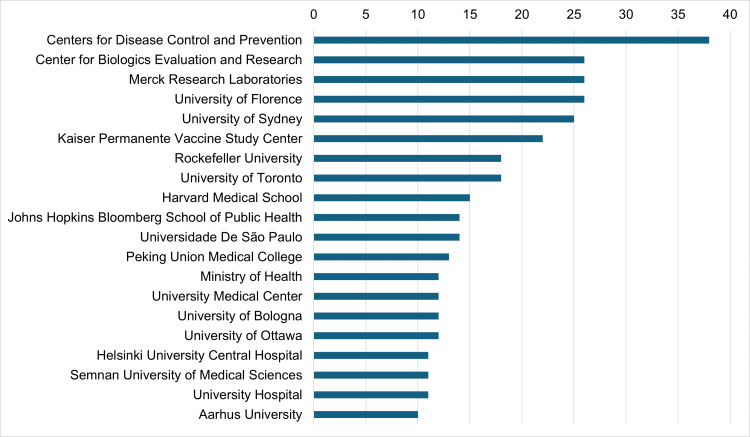
Major institutions involved in publications on mumps vaccine

The majority of documents included in the analysis were authored by researchers from developed nations like the United States and the United Kingdom. The top three institutions involved in mumps-related research were: Centers for Disease Control And Prevention (38 documents) - the national public health agency of the United States; Center for Biologics Evaluation and Research (CBER) (26 documents) - an organization within the Food & Drug Administration (FDA), which is an agency under the Department of Health and Health Services under the Federal Government of the United States which regulates biological products for human use under the various laws in the US; and Merck Research Laboratory Laboratories (26 documents) under Merck Pharmaceutical Company which is involved in research and development of biopharmaceuticals in the US (Figure 5).

Distribution of Countries Involved in the Publication on the Mumps Vaccine

**Figure 6:**
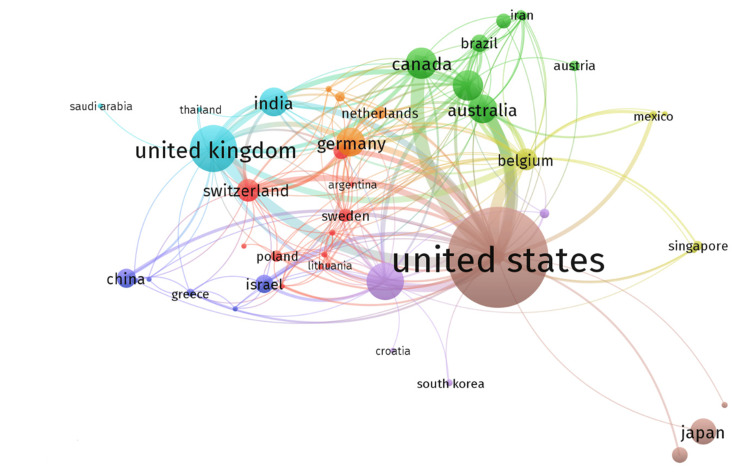
Outstanding countries contributing to publications on mumps vaccine

The countries with the highest scientific production were the United States (1037), the United Kingdom (266), and Italy (256) (Figure 6).

**Figure 7:**
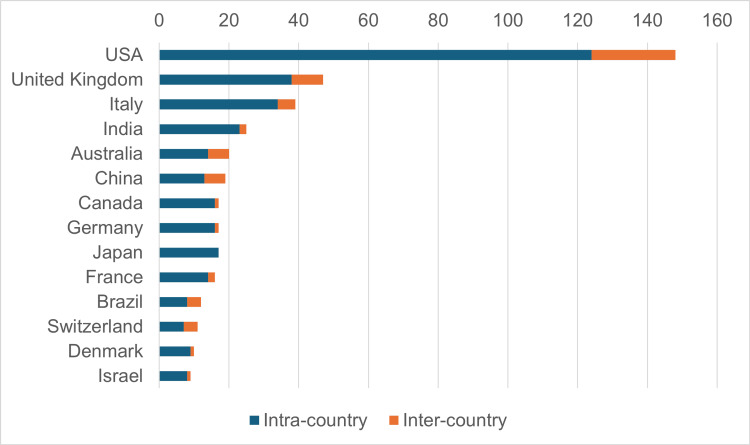
Outstanding countries with corresponding author of publication on mumps vaccine

The top three countries based on the number of citations received for the publication of documents related to the mumps vaccine were the United States (n=6855), the United Kingdom (3034), and Italy (988), with an average article citation of 46.30, 64.60, and 25.30, respectively. The outstanding countries based on corresponding authors of publications were the United States, the United Kingdom, and Italy, with 148, 47, and 39 articles; 24, nine, and five inter-country publications; and 124, 38, and 34 intra-country publications, respectively. Countries with moist climates around the world placed around the equator, especially in Africa, Latin America, and small island nations in the Pacific, are the epicenter of the mumps burden. Still, the research output from these nations is limited (Figure 7).

Health organizations should work actively in the direction of funding mumps-related research activities to uncover the hidden epidemiological importance of mumps in order to create effective control and prevention strategies.

Distribution of Sources of Publications on Mumps Vaccine

**Figure 8:**
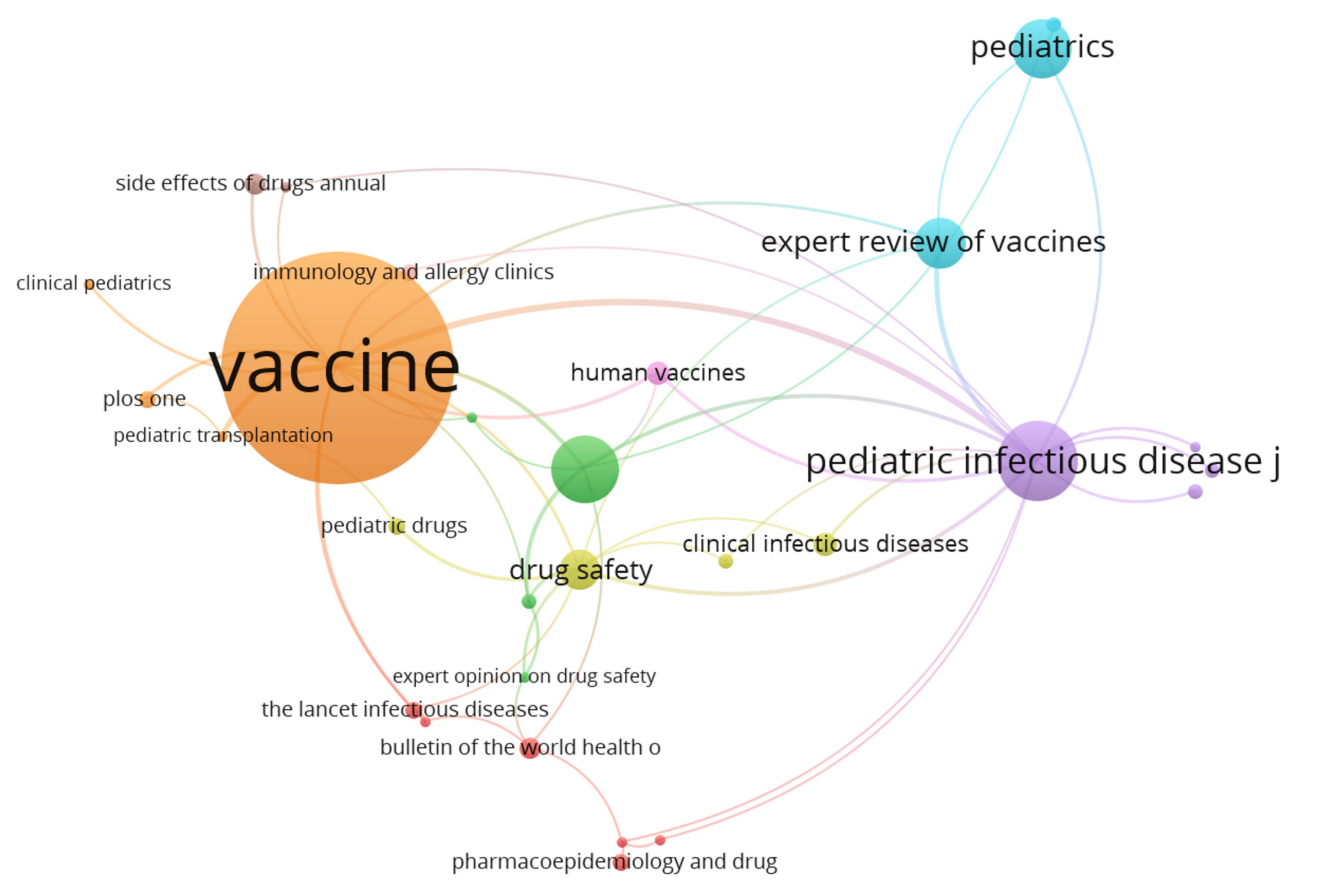
Outstanding sources contributing to publications on mumps vaccine

Journals like Vaccine from Elsevier, Paediatric Infectious Disease Journal (Pediatr Infect Dis J) from Wolter Kluwer, and Human Vaccines and Immunotherapeutics by Taylor & Francis were the top three sources with 90, 27, and 22 articles, respectively. The sources with the highest local citations (from reference lists) were Vaccine from Elsevier, Drug Safety, which is the official journal of the International Society of Pharmacovigilance (ISoP), Paediatrics by the American Academy of Paediatrics, with 2940, 1801, and 1555 citations, respectively. Vaccine from Elsevier, Paediatrics by American Academy of Paediatrics, and Paediatric Infectious Disease Journal (Pediatr Infect Dis J) from Wolter Kluwer were the top three sources on the basis of impact with an index of 29, 17, and 15; a g-index of 51, 19, and 27; an m-index of 0.853, 0.515, and 0.417; total number of citations of 2940, 1555, and 1201; and publication year starting from 1991, 1992, and 1989, respectively (Figure 8).

Distribution of Keywords in Publications on Mumps Vaccine

**Figure 9:**
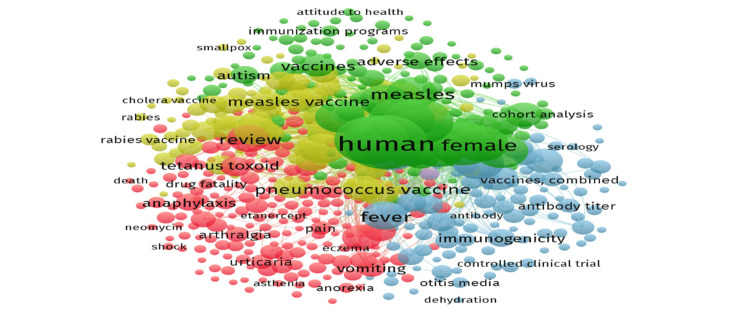
Dominant keywords in publications on mumps vaccine

'Human', 'vaccination', and 'measles, mumps, rubella vaccine' were the top three keywords used in the studies, with 677, 655, and 575 occurrences, respectively (Figure 9).

Inter-Country Collaboration in Publications on the Mumps Vaccine

**Figure 10:**
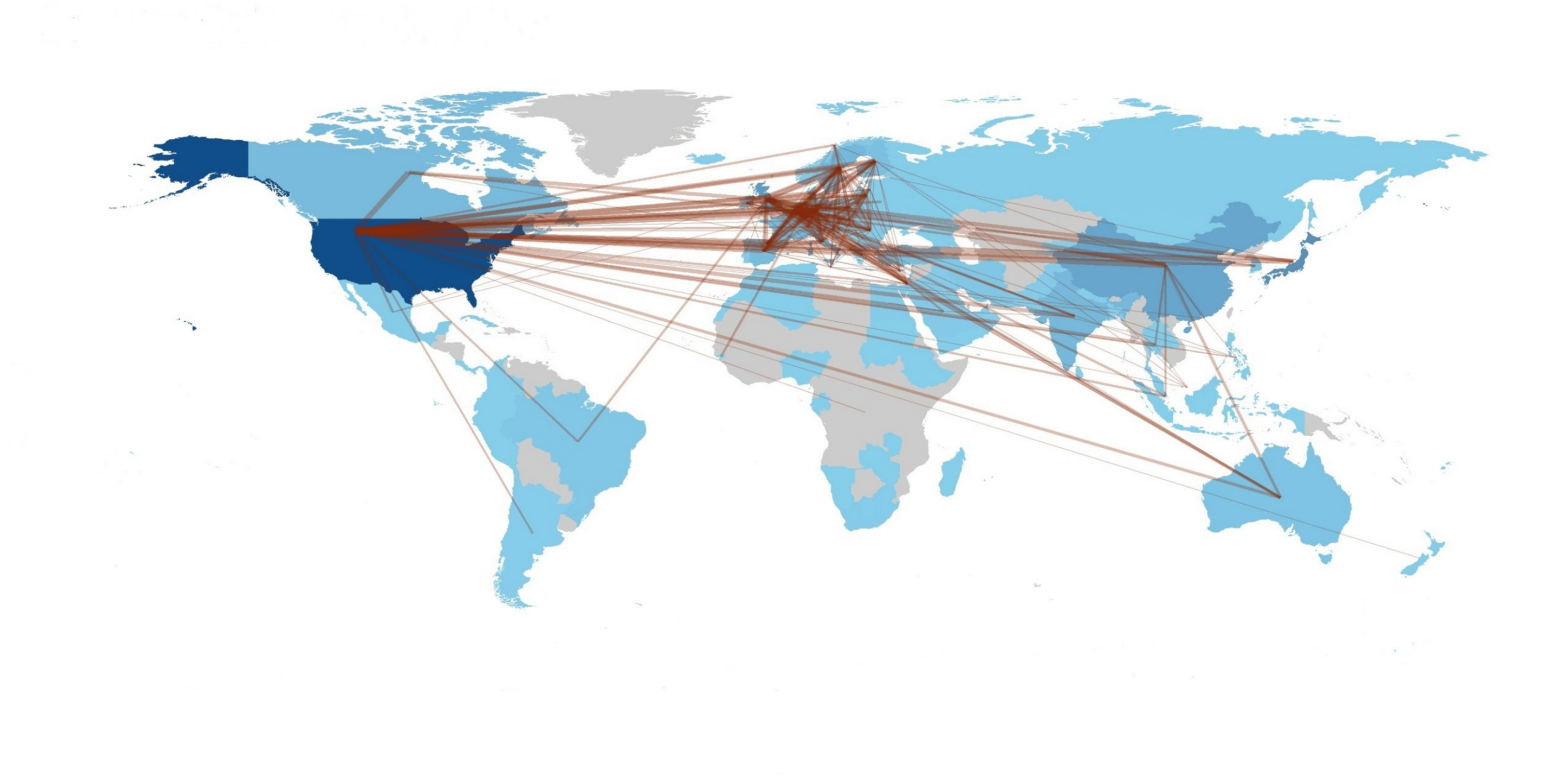
Worldwide collaborations of researchers of publications on mumps vaccine

The top three inter-country collaborations on mumps vaccine-based studies were between the United States and Canada, the United States and Belgium, and the United States and the United Kingdom, with 13, 11, and 10 collaborations, respectively (Figure 10).

Discussion

All original research documents related to the mumps vaccine from 1972 to 2021 (50 years) are covered in this bibliometric analysis. The study was able to describe research on the mumps vaccine more comprehensively and descriptively. The research publications were widespread in both developed and developing countries. Researchers from around the globe have worked on a wide range of topics related to the mumps vaccine, like vaccine development, vaccine strains, vaccine safety, vaccine-related research and development, etc. No bibliometric analysis has been done on such a large scale, analyzing the documents from the last 50 years. Bibliometric analysis gives an overview of the research volume, the involvement of various agencies, author collaborations, and inter/ intra-country collaborations. This study revealed around 699 articles related to mumps vaccine-related studies in Scopus, with an average number of citations per document being 33.87. There has been marked growth in publications since the beginning of the 21st century, and a peak in research documents published on the mumps vaccine was seen in 2015. The annual growth rate for the production of documents is 6.38%. The increase in publications from the early 2000s can be attributed to a significant increase in mumps outbreaks, concerns over vaccine efficacy and safety, waning immunity after vaccination, and attempts to develop newer vaccines with enhanced immune responses [30]. However, causality cannot be assessed due to a lack of an in-depth analysis of available literature.

In recent years, there has been a cost-benefit analysis for the inclusion of the mumps vaccine into the immunization schedule of various countries. Several researchers have tried to analyze the cost of replacing the MR vaccine with the MMR vaccine or implementing a third adult dose into the schedule where two doses of MMR are already being given [31,32]. There is increased research in the field of immunology of the mumps vaccine. Immunology can be defined as the study of the immune response and cellular and molecular events that occur when the host body's immune system identifies a microbe or a foreign body (micro and macromolecules). Vaccines and immunology have a close connection as the vaccines contain the antigenic substances, either in the killed form or attenuated form, that induce an immune response in the individual and prevent the individual from getting the disease, or even if the disease occurs, it shall appear in its mild form with an attenuated clinical manifestation [33,34]. Most studies are multiauthor, with around 15% of documents having a single author. Several citations and their various characteristics were analyzed in this study, which helps analyze mumps vaccine-related research documents in multiple ways. The top three globally cited studies had more than 2500 citations. The Centers for Disease Control (CDC), a federal agency of the United States, has the highest number of documents related to the mumps vaccine, followed by the Center for Biologics Evaluation and Research (CBER), which is an organization within the Food & Drug Administration (FDA), another federal agency in the United States. The involvement of health agencies, research institutes, and pharmaceutical companies in developing and producing biopharmaceuticals like vaccines in the mumps vaccine-related studies is visibly high in developed countries compared to low- and middle-income countries. Population giants like India and China are not that active in vaccine research.

Countries of the 'Global North', such as the United States, the United Kingdom, and Italy, had the highest number of published studies and corresponding authors. According to Bradford's analysis, only 14 journals were in the core zone. Around one-third of all documents on the mumps vaccine were published in these 14 journals, indicating that a limited number of journals have a significant concentration of publications. The study indicates a need to establish research collaboration and enhance inter-country cooperation with the researchers and experts of different countries [35]. The journals with the majority of published articles on mumps vaccine-related studies have a hybrid open-access publication model. This may lead to high-quality local research that is not published and noticed. The study results reveal that three keywords were used: human, vaccination, and the measles, mumps, rubella vaccine. This study gives an overview of research on the mumps vaccine that has been done in the last 50 years. These 50 years have seen a changing dynamic of infection, virulence, vaccination strategy, and vaccine development of the mumps vaccine. This study will be valuable for medical practitioners, microbiologists, epidemiologists, public health experts, policymakers, and researchers. Scientists and researchers working on new vaccine development will be helped by this study, focusing on various domains related to mumps vaccine-related research. Research on the mumps vaccine has been very limited. The scientific society is more involved in developing new vaccines for other communicable and vector-borne diseases. Mumps as a disease with minimal risk of complication and under-reporting by governments regarding the number of mumps cases or mumps outbreaks can be considered a factor for this thin interest in mumps vaccine-related research. Large countries like India are reporting single-digit mumps cases with no reporting regarding the ongoing outbreaks, making mumps a neglected disease, and thus, research activity on the mumps vaccine and mumps as a disease as a whole is less [36-38]. Research on the mumps vaccine should be encouraged through scientific and financial collaborations. The study gives a basic idea of the current status and trends in mumps vaccine-related research. This study is the first bibliometric analysis of publications on the mumps vaccine to identify the most prolific authors, institutions, countries, and journals in the Scopus database.

Limitation

The aim of the study was limited to all documents published and indexed in the Scopus database and related to the mumps vaccine. A comparison between different databases is out of the scope of this study. However, the documents indexed in other databases may differ significantly from the current research, and the analysis of results from those databases may vary from the current study. We were not able to consider access to different language papers during the analysis. We also did not consider potential self-citations, citation-lag bias, or potential inflation from large collaborative consortia in this study. We excluded the articles published in the last three years to remove the possibility of excluding articles from the databases.

## Conclusions

The global scientific output on mumps vaccine-related studies was estimated and evaluated by analyzing the records from the journals indexed in the Scopus database. The study helped identify the top authors, journals, institutions, and countries. It also analyzed the distribution of articles globally and the interaction between researchers. Several articles have provided an open-access policy so that other researchers can utilize their work on the mumps vaccine. Newer software like Biblioshiny, a Bibliometric package of R software, and VOSViewer help conduct bibliometric analysis on any topic with easy construction and visualization of the bibliometric web. This software has also helped me to understand the research trends regarding publication rate and subject. The study has tried to summarise the pattern of mumps vaccine-related research and will help offer a valuable perspective and direction to future studies. There exists a large ocean of unexplored scientific data to work on.
